# Association Between MicroRNAs Polymorphisms and Risk of Ischemic Stroke: A Meta-Analysis in Chinese Individuals

**DOI:** 10.3389/fnagi.2018.00082

**Published:** 2018-03-28

**Authors:** Chen-Xi Li, Hong Weng, Jun Zheng, Zhi-He Feng, Jian-Lin Ou, Wei-Jing Liao

**Affiliations:** ^1^Department of Rehabilitation Medicine, Zhongnan Hospital of Wuhan University, Wuhan, China; ^2^Center for Evidence-Based and Translational Medicine, Zhongnan Hospital of Wuhan University, Wuhan, China; ^3^Department of Urology, Zhongnan Hospital of Wuhan University, Wuhan, China

**Keywords:** miR-146a, miR-149, miR-196a2, miR-499, polymorphism, ischemic stroke, risk, meta-analysis

## Abstract

**Objective:** Previous studies have demonstrated that some single-nucleotide polymorphisms (SNPs) in miRNAs are related to the risk of ischemic stroke (IS), but the conclusions are still controversial and inconclusive. We performed this meta-analysis to further assess the association between miR-146a C>G (rs2910164), miR-149 T>C (rs2292832), miR-196a2 T>C (rs11614913), miR-499 A>G (rs3746444) and risk of IS in Chinese individuals.

**Methods:** Relevant studies were identified in the databases of PubMed, Embase. The strength of correlation between microRNAs polymorphisms and IS risk was assessed by odds ratios (ORs) and 95% confidence intervals (95% CIs) under five genetic models.

**Results:** 5 studies, containing 2,632 cases and 3,191 controls, were included in this meta-analysis. The overall results of meta-analysis indicated that there were no significant association between miR-146a C>G (rs2910164), miR-149 T>C (rs2292832), miR-196a2 T>C (rs11614913), and the IS risk in the overall analyses. MiR-499 A>G (rs3746444) was associated with an increased IS risk under allele model (OR = 1.30, 95% CI = 1.02–1.66), heterozygous model (OR = 1.35, 95% CI = 1.01–1.79) and dominant model (OR = 1.36, 95% CI = 1.02–1.80) in Chinese. The sensitivity analysis results of these four polymorphisms were similar to the overall results.

**Conclusion:** MiR-499 A>G (rs3746444) G allele and AG, AG + AA genotype might be risk factors of IS in Chinese. No significant association was observed between miR-146a C>G (rs2910164), miR-149 T>C (rs2292832), miR-196a2 T>C (rs11614913), and IS risk. The associations may be different due to geographical factors of China. More explorations in more diverse geographically regions with large sample size are expected to further verify the findings in the future.

## Introduction

Neurological disorders are the second leading cause of death and a major cause of functional disability worldwide, among which stroke accounted for the highest proportion, imposing a huge burden for the whole society as well as the healthcare system (GBD Neurological Disorders Collaborator Group, 2017). Ischemic stroke (IS) occupies approximately 85% of all types of strokes (Kotlega et al., 2017). The risk factors of IS include hypertension, smoking, waist-to-hip ratio, diet, diabetes mellitus, etc. (O'Donnell et al., 2010). Moreover, a large range of genetic factors mingled with environmental factors stimulated the complex disease, and it is indicated that genetic ones play a significant role in the cause of IS (Boehme et al., 2017; Pan et al., 2017; Xiang et al., 2017).

MicroRNAs (miRNAs) are non-coding, endogenous ~22 nt RNAs, which can induce either translational repression or mRNA degradation, and regulate post-transcriptional gene expression by binding to the 3′-untranslated region (3′-UTR) of target genes (Bartel, 2009). The most abundant form of DNA variation in the human genome are single nucleotide polymorphisms (SNPs), which can potentially change various biological processes by affecting the maturation process or target selection of miRNAs (Duan et al., 2007). Previous studies have demonstrated that miR-146a G, miR-149 T, miR-196a2 C, miR-499 G alleles are possible genetic predisposing factors (Jeon et al., 2012; Min et al., 2012; Park et al., 2012; Wang et al., 2012). These four SNPs in miRNAs (miR-SNPs) can influence vascular damage responses, and regulate miRNA targets related to thrombosis and inflammation pathways in the circulation system (Luthra et al., 2008; El Gazzar et al., 2011; Yang et al., 2012; Wu et al., 2013). Among them, miR-146a is closely related to regulation of tumor necrosis factor-α (TNF-α) (El Gazzar et al., 2011), meanwhile miR-149 can regulate the expression of MTHFR (Wu et al., 2013), miR-196a2 can target annexin A1 (Luthra et al., 2008), and miR-499 can affect the inflammatory reaction through modulating C-reactive protein (CRP) (Yang et al., 2012) respectively. Several studies aimed to explore the influence of these four SNPs on IS risk, but the conclusions are still controversial and inconclusive. We performed this meta-analysis to further assess the association between miR-146a C>G (rs2910164), miR-149 T>C (rs2292832), miR-196a2 T>C (rs11614913), miR-499 A>G (rs3746444) and risk of IS in Chinese individuals, which may be likely to be utilized as clinical indexes to evaluate the risk of occurrence, development and responses to the treatments of IS.

## Materials and methods

### Search strategy

We conducted a comprehensive literature search of PubMed, Embase up to November 30, 2017. The searched terms are as follows: [“ischemic stroke” AND (“microRNA” OR “miRNA” OR “miR”) AND (“polymorphism” OR “SNP” OR “mutation” OR “variant”)]. In order to identify other studies which may be relevant with this from references, all studies were retrieved in the manual way. We performed the meta-analysis according to Preferred Reporting Items for Systematic Reviews and Meta-Analyses (PRISMA) statement in reporting meta-analysis.

### Eligibility criteria

Eligible studies should conform with the following criteria: (1) the exposure was microRNAs polymorphisms; (2) populations were divided into case and control groups, consisting of IS and without IS respectively; (3) the outcome was incident of IS; (4) the study design was accordance with case-control study; (5) study provided sufficient published data to calculate the odds ratios (ORs) with corresponding 95% confidence intervals (CIs). The exclusion conditions include: (1) repeated publications (studies recently published or with more participants were included); (2) abstract, reviews, meta-analysis, case reports and comments. (3) animal model research. (4) not about Chinese individuals; (5) not published in English.

### Data extraction

Information was extracted from all included studies for analysis, including first author's name, published year, country, ethnicity, source of controls, genotype frequency of case and control, age, genotyping method, and HWE for controls by two independent researchers (Li and Weng). Discrepancy was resolved by referring to original studies in discussion with a third reviewer (Zheng).

### Statistical analysis

Chi-square test was used to determine Hardy-Weinberg equilibrium (HWE) in the control groups, which could be considered disequilibrium when *P* value is less than 0.05. The strength of correlation between microRNAs polymorphisms and IS risk was assessed by ORs and 95% confidence intervals (95% CIs) under five genetic models. Heterogeneity between the studies was evaluated by both the Cochran's Q statistic and the *I*^2^ statistic (Higgins, 2008). *P* < 0.10 and/or *I*^2^ > 50% indicated significant heterogeneity, and random-effects model (the DerSimonian and Laird method) was applied for ORs calculation; otherwise, fixed-effects model using the Mantel-Haenszel was utilized. The sensitivity analysis was performed according to HWE status of controls (Thakkinstian et al., 2005). Furthermore, Begg's funnel plot and Egger's test were used to inspect potential publication bias among included studies. The statistical significance of the ORs was determined by *Z*-test, with *P* < 0.05 suggesting a significant difference. All statistical analyses were conducted with the software Stata 14.0.

## Results

### Characteristics of included studies

**Figure 1:**
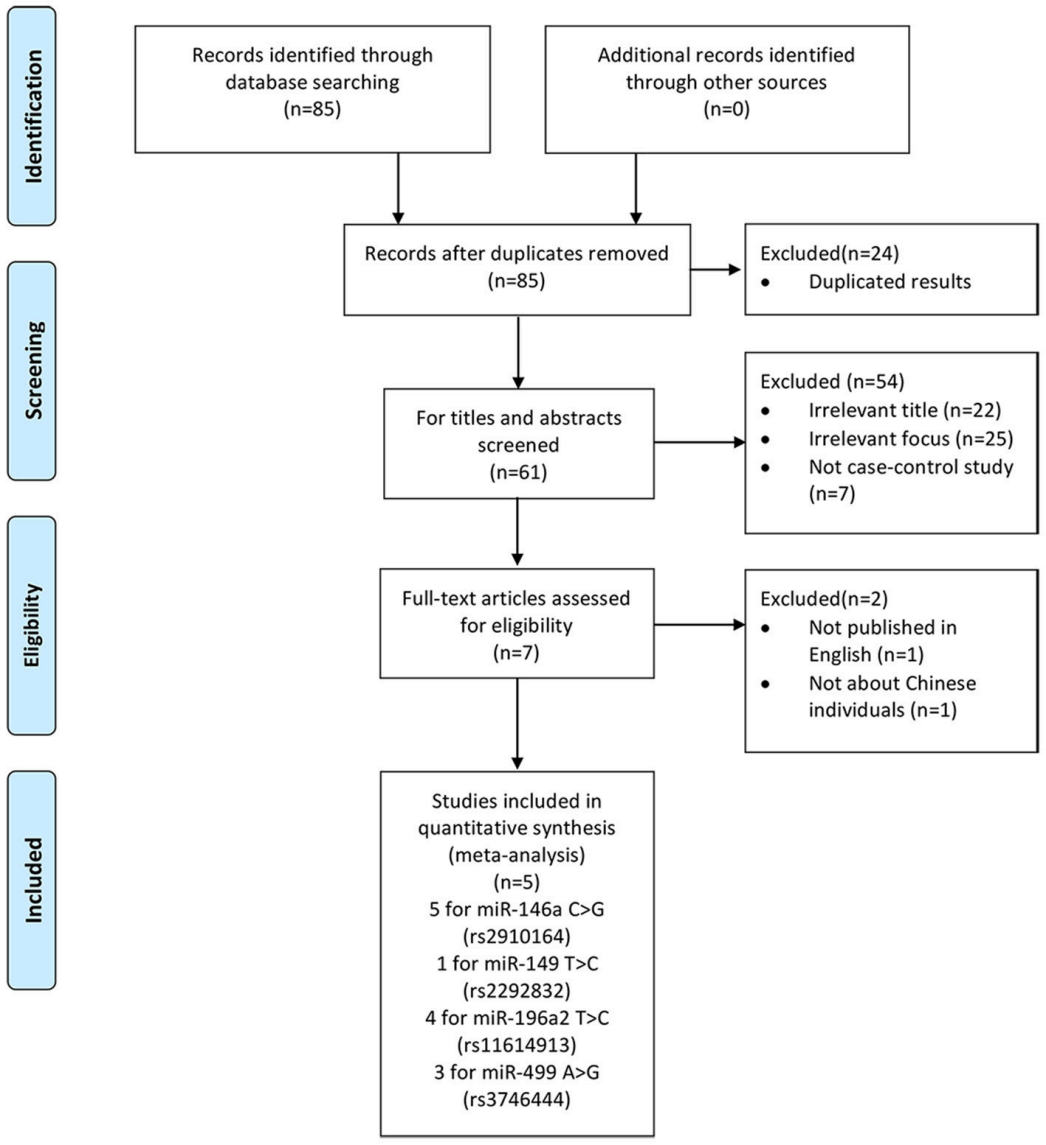
Flow chart of study selection process.

We yielded 85 papers initially and 5 studies, containing 2,632 cases and 3,191 controls, were included in this meta-analysis finally. Figure 1 shows the progress of study selection. Among these studies, five studies focused on miR-146a G>C (rs2910164) (Liu et al., 2014; Zhu et al., 2014; Huang et al., 2015; Qu et al., 2016; Luo et al., 2017), one study focused on miR-149 T>C (rs2292832) (Luo et al., 2017), four studies focused on miR-196a2 T>C (rs11614913) (Liu et al., 2014; Zhu et al., 2014; Huang et al., 2015; Luo et al., 2017) and three studies focused on miR-499 A>G (rs3746444) (Liu et al., 2014; Huang et al., 2015; Luo et al., 2017). The characteristics of the included studies are presented in Table 1.Among them, despite one study (Qu et al., 2016) in miR-146a G>C (rs2910164), one study (Huang et al., 2015) in miR-499 A>G (rs3746444), all conformed with HWE.

**Table 1:** Major characteristics of included studies.

**First author**	**Year**	**Country**	**Ethnicity**	**Source of control**	**Age (Case/Control)**	**Sample size (Case/Control)**	**Genotyping method**	***P* for HWE**
**miR-146a C>G (rs2910164)**								
Liu	2014	China	Asian	Healthy	67.52 ± 10.29/66.34 ± 11.07	296/391	PCR-RFLP	0.650
Zhu	2014	China	Asian	Healthy	61.62 ± 0.986/62.05 ± 0.982	368/381	PCR-LDR	0.952
Huang	2015	China	Asian	Healthy	63 (54, 70)/61 (54, 68)	531/531	Taqman	0.107
Qu	2016	China	Asian	Healthy	61.30 ± 9.40/59.50 ± 8.50	1,139/1,585	PCR-LDR	<0.01
Luo	2017	China	Asian	Healthy	60.70 ± 12.33/60.17 ± 10.32	298/303	PCR	0.672
**miR-149 T>C (rs2292832)**								
Luo	2017	China	Asian	Healthy	60.70 ± 12.33/60.17 ± 10.32	298/303	PCR	0.447
**miR-196a2 T>C (rs11614913)**								
Liu	2014	China	Asian	Healthy	67.52 ± 10.29/66.34 ± 11.07	296/391	PCR-RFLP	0.060
Zhu	2014	China	Asian	Healthy	61.62 ± 0.986/62.05 ± 0.982	368/381	PCR-LDR	0.384
Huang	2015	China	Asian	Healthy	63 (54, 70)/61 (54, 68)	531/531	Taqman	0.856
Luo	2017	China	Asian	Healthy	60.70 ± 12.33/60.17 ± 10.32	298/303	PCR	0.385
**miR-499 A>G (rs3746444)**								
Liu	2014	China	Asian	Healthy	67.52 ± 10.29/66.34 ± 11.07	296/391	PCR-RFLP	0.170
Huang	2015	China	Asian	Healthy	63 (54, 70)/61 (54, 68)	531/531	Taqman	<0.01
Luo	2017	China	Asian	Healthy	60.70 ± 12.33/60.17 ± 10.32	298/303	PCR	0.132

*HWE, Hardy-Weinberg equilibrium; PCR-RFLP, polymerase chain reaction–restriction fragment length polymorphism; PCR-LDR, polymerase chain reaction-ligase detection reaction; PCR, polymerase chain reaction*.

### Heterogeneity test

The result of between-study heterogeneity of genetic models of miR-146a G>C (rs2910164) polymorphism was significant (Table 2) under three models (G vs. C, *I*^2^ = 60.1, *P* = 0.04; GG vs. CC, *I*^2^ = 61.4, *P* = 0.04; GG vs. CG + CC, *I*^2^ = 50.2, *P* = 0.09). The similar trend can be observed in miR-499 A>G (rs3746444) polymorphism under allele model (G vs. A, *I*^2^ = 52.8, *P* = 0.12), heterozygous model (AG vs. AA, *I*^2^ = 54.3, *P* = 0.11) and dominant model (AG + GG vs. AA, *I*^2^ = 56.1, *P* = 0.10), thus random-effects model was used for meta-analysis.

**Table 2:** Overall and sensitivity analyses of miR-146a C>G (rs2910164) and risk of IS.

**Genetic model**	**Subgroup**	**No. of studies**	**Meta-analysis**		***P* for Egger's test**	**Heterogeneity**	
			**OR (95%CI)**	***P*-value**		***I*^2^ (%)**	***P*-value**
G vs. C	Overall	5	0.99 (0.87–1.12)	0.87	0.77	60.1	0.04
	HWE-Yes	4	0.99 (0.82–1.19)	0.88		69.8	0.02
GG vs. CC	Overall	5	0.99 (0.74–1.31)	0.92	0.85	61.4	0.04
	HWE-Yes	4	0.98 (0.65–1.48)	0.94		70.8	0.02
CG vs. CC	Overall	5	0.99 (0.88–1.11)	0.85	0.50	0	0.46
	HWE-Yes	4	1.01 (0.86–1.18)	0.93		13.7	0.32
CG + GG vs. CC	Overall	5	0.99 (0.89–1.11)	0.87	0.31	39.3	0.16
	HWE-Yes	4	0.99 (0.80–1.23)	0.94		53.5	0.09
GG vs. CG + CC	Overall	5	0.99 (0.79–1.24)	0.94	0.85	50.2	0.09
	HWE-Yes	4	0.99 (0.71–1.37)	0.94		62.6	0.05

*OR, odds ratio; CI, confidence interval; HWE, Hardy-Weinberg equilibrium*.

The fixed-effects model was used to calculate ORs for miR-196a2 T>C (rs11614913) polymorphism due to the low heterogeneity under all genetic models. No evidence of significant heterogeneity was detected in the heterozygous model (CG vs.CC, *I*^2^ = 0, *P* = 0.46) and dominant model (CG + GG vs. CC, *I*^2^ = 39.3, *P* = 0.16) of miR-146a G>C (rs2910164). So does the homozygous model (GG vs. AA, *I*^2^ = 18.6, *P* = 0.27) and recessive model (GG vs. AG + AA, *I*^2^ = 17.7, *P* = 0.27) of miR-499 A>G (rs3746444) polymorphism.

The vast territory of China consists of different climate zones as well as environmental differences are known to exist between the Northern and Southern, the coastal and inland, mountains and plains. The meta-analysis verified our result and also indicated that geographical factors may be one of the main causes of high heterogeneity.

### MiR-146a C>G (rs2910164) and the risk of is

There was no significant association between this SNP and the IS risk in the overall analyses. (G vs. C: OR = 0.99, 95% CI = 0.87–1.12; GG vs. CC: OR = 0.99, 95% CI = 0.74–1.31; CG vs. CC: OR = 0.99; 95% CI = 0.88–1.11; CG + GG vs. CC: OR = 0.99, 95% CI = 0.89–1.11; GG vs. CG + CC: OR = 0.99, 95% CI = 0.79–1.24, Table 2).

**Figure 2:**
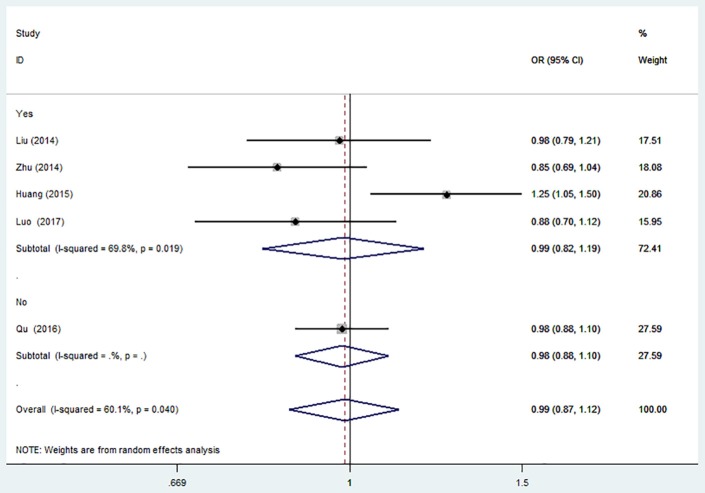
Forest plot for the association between miR-146a C>G (rs2910164) polymorphism and ischemic stroke risk in allele genetic model.

The results of sensitivity analysis were similar to the overall analyses (Figure 2).

### MiR-149 T>C (rs2292832) and the risk of is

**Figure 3:**
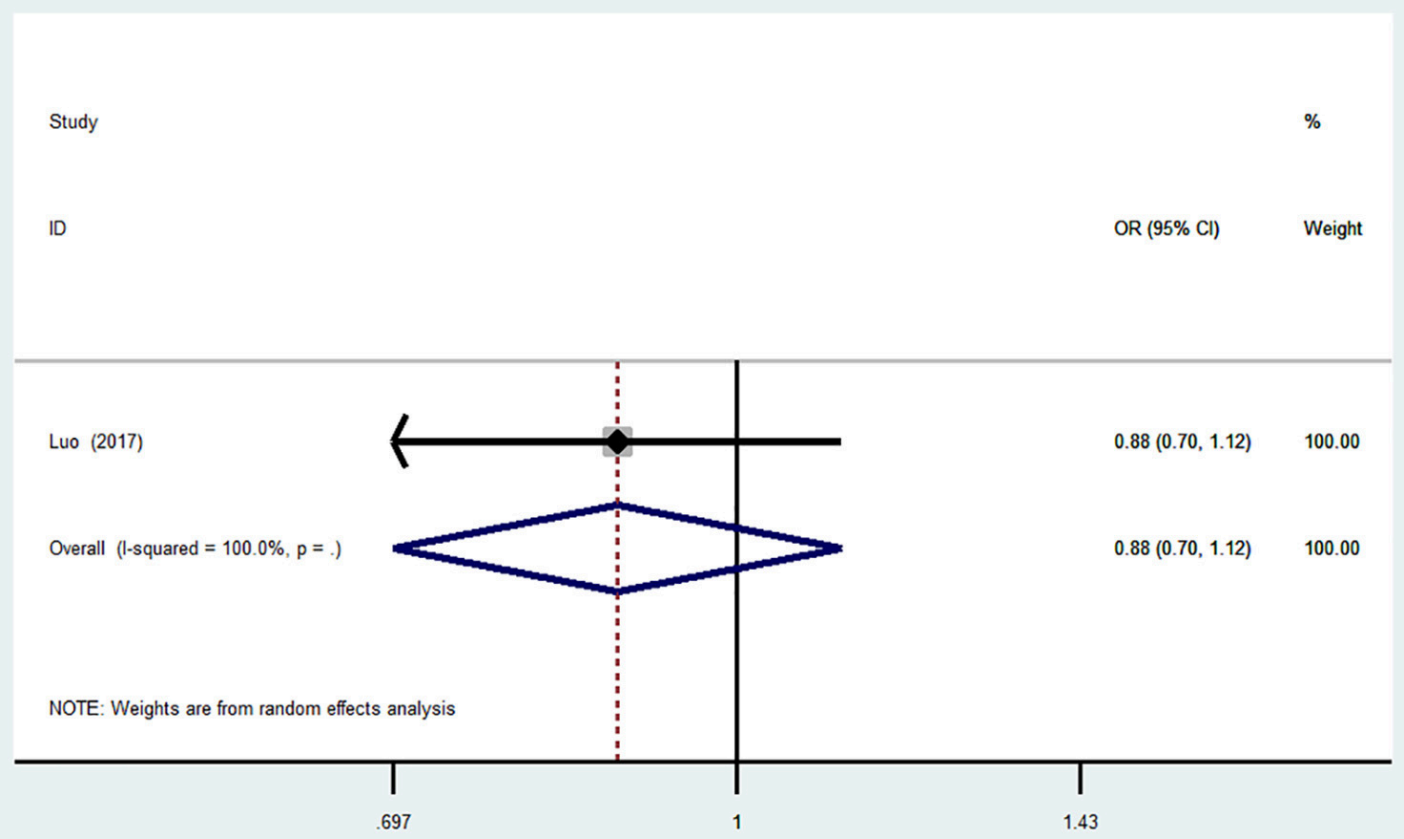
Forest plot for the association between miR-149 T>C (rs2292832) polymorphism and ischemic stroke risk in allele genetic model.

No significant correlation was found in the overall analyses or sensitive analyses in any one of these models (C vs. T: OR = 0.88, 95% CI = 0.70–1.12; CC vs. TT: OR = 0.80, 95% CI = 0.49–1.31; TC vs. TT: OR = 0.86; 95% CI = 0.61–1.22; TC + CC vs. TT: OR = 0.85, 95% CI = 0.61–1.17; CC vs. TC + TT: OR = 0.87, 95% CI = 0.55–1.37, Table 3, Figure 3).

**Table 3:** Overall analyses of miR-149 T>C (rs2292832) and risk of IS.

**Genetic model**	**Subgroup**	**No. of studies**	**Meta-analysis**		***P* for Egger's test**	**Heterogeneity**	
			**OR (95%CI)**	***P*-value**		***I*^2^ (%)**	***P*-value**
C vs. T	Overall	1	0.88 (0.70–1.12)	0.30	–	–	–
CC vs. TT	Overall	1	0.80 (0.49–1.31)	0.38	–	–	–
TC vs. TT	Overall	1	0.86 (0.61–1.22)	0.40	–	–	–
TC + CC vs. TT	Overall	1	0.85 (0.61–1.17)	0.30	–	–	–
CC vs. TC + TT	Overall	1	0.87 (0.55–1.37)	0.54	–	–	–

*OR, odds ratio; CI, confidence interval*.

### MiR-196a2 T>C (rs11614913) and the risk of is

**Figure 4:**
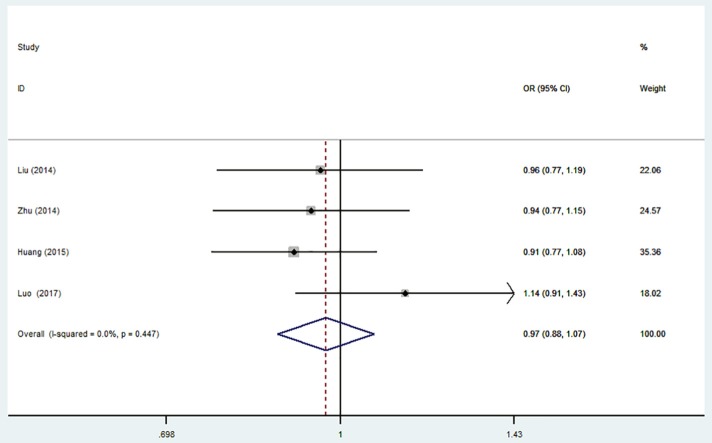
Forest plot for the association between miR-196a2 T>C (rs11614913) polymorphism and ischemic stroke risk in allele genetic model.

There was no significant association between this SNP and the IS susceptibility under all genetic models (C vs. T: OR = 0.97, 95% CI = 0.88–1.07; CC vs. TT: OR = 0.93, 95% CI = 0.76–1.15; TC vs. TT: OR = 0.97; 95% CI = 0.82–1.15; TC + CC vs. TT: OR = 0.96, 95% CI = 0.82–1.13; CC vs. TC + TT: OR = 0.96, 95% CI = 0.80–1.14, Table 4, Figure 4).

**Table 4:** Overall analyses of miR-196a2 T>C (rs11614913) and risk of IS.

**Genetic model**	**Subgroup**	**No. of studies**	**Meta-analysis**		***P* for Egger's test**	**Heterogeneity**	
			**OR (95%CI)**	***P*-value**		***I*^2^ (%)**	***P*-value**
C vs. T	Overall	4	0.97 (0.88–1.07)	0.56	0.21	0	0.45
CC vs. TT	Overall	4	0.93 (0.76–1.15)	0.52	0.44	0	0.45
TC vs. TT	Overall	4	0.97 (0.82–1.15)	0.76	0.61	0	0.56
TC + CC vs. TT	Overall	4	0.96 (0.82–1.13)	0.64	0.59	0	0.73
CC vs. TC + TT	Overall	4	0.96 (0.80–1.14)	0.61	0.83	49.1	0.12

*OR, odds ratio; CI, confidence interval*.

### MiR-499 A>G (rs3746444) and the risk of is

**Figure 5:**
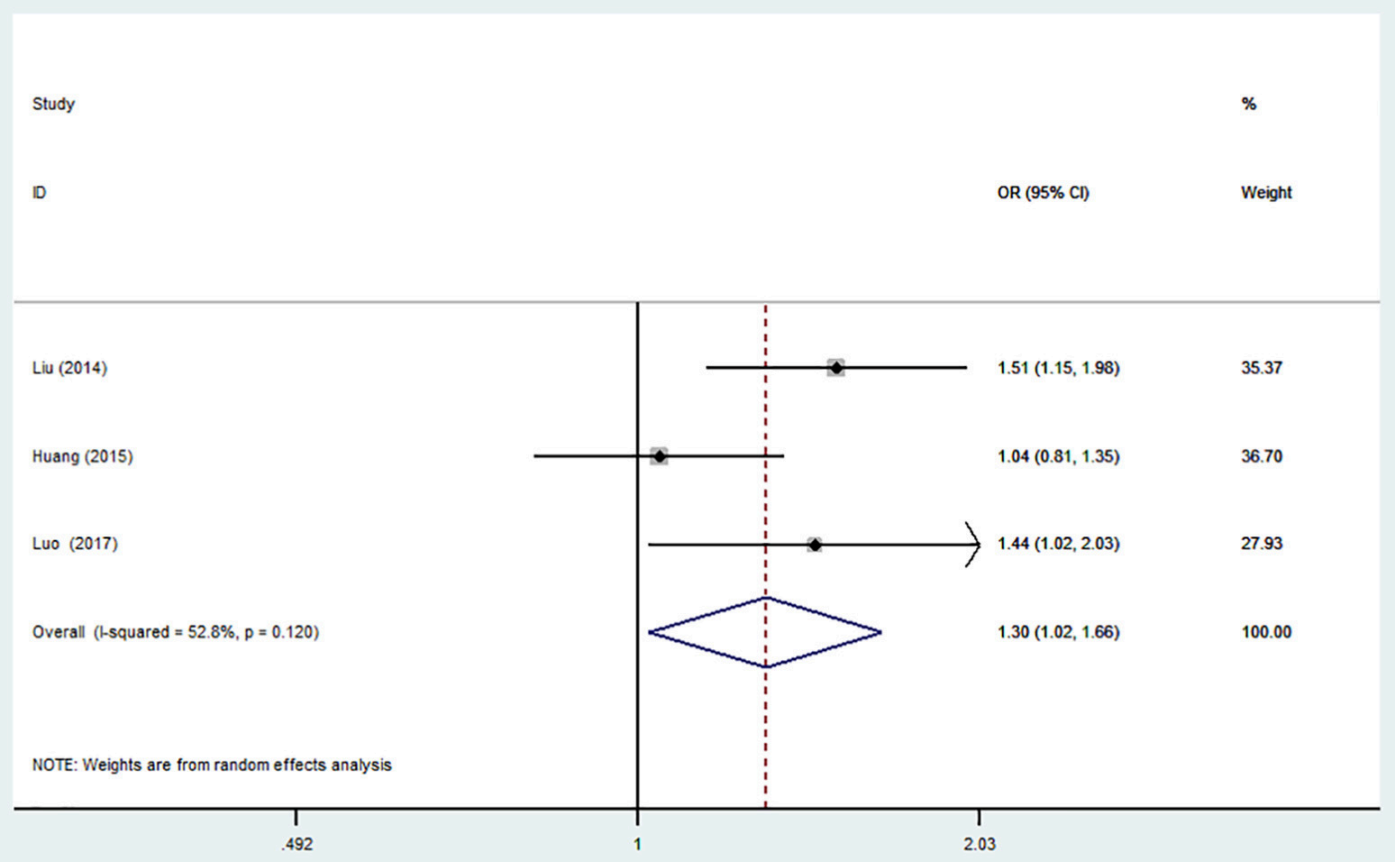
Forest plot for the association between miR-499 A>G (rs3746444) polymorphism and ischemic stroke risk in allele genetic model.

The overall analysis showed that the miR-499 A>G (rs3746444) polymorphism increased the risk of IS under allele model (G vs. A, OR = 1.30, 95% CI = 1.02–1.66), heterozygous model (AG vs. AA, OR = 1.35; 95% CI = 1.01–1.79) and dominant model (AG + GG vs. AA: OR = 1.36, 95% CI = 1.02–1.80). No significant association was observed between miR-499 A>G (rs3746444) polymorphism and risk of IS under homozygous model (GG vs. AA: OR = 1.02, 95% CI = 0.71–1.46) and recessive model (GG vs. AG + AA, OR = 1.51, 95% CI = 0.82–2.75) (Table 5, Figure 5).

**Table 5:** Overall and sensitivity analyses of miR-499 A>G (rs3746444) and risk of IS.

**Genetic model**	**Subgroup**	**No. of studies**	**Meta-analysis**		***P* for Egger's test**	**Heterogeneity**	
			**OR (95%CI)**	***P*-value**		***I*^2^ (%)**	***P*-value**
G vs. A	Overall	3	1.30 (1.02–1.66)	<0.05	0.67	52.8	0.12
	HWE-Yes	2	1.48 (1.20–1.83)	<0.05		0	0.84
GG vs. AA	Overall	3	1.69 (0.92–3.10)	0.09	–	18.6	0.27
	HWE-Yes	2	1.69 (0.92–3.10)	0.09		18.6	0.27
AG vs. AA	Overall	3	1.35 (1.01–1.79)	<0.05	0.17	54.3	0.11
	HWE-Yes	2	1.56 (1.21–2.02)	<0.05		0	0.67
AG + GG vs. AA	Overall	3	1.36 (1.02–1.80)	<0.05	0.36	56.1	0.10
	HWE-Yes	2	1.58 (1.23–2.02)	<0.05		0	0.93
GG vs. AG + AA	Overall	3	1.51 (0.82–2.75)	0.19	–	17.7	0.27
	HWE-Yes	2	1.51 (0.82–2.75)	0.19		17.7	0.27

*OR, odds ratio; CI, confidence interval; HWE, Hardy-Weinberg equilibrium*.

Sensitivity analysis was conducted. However, no change of the result was detected.

### Publication bias

**Figure 6:**
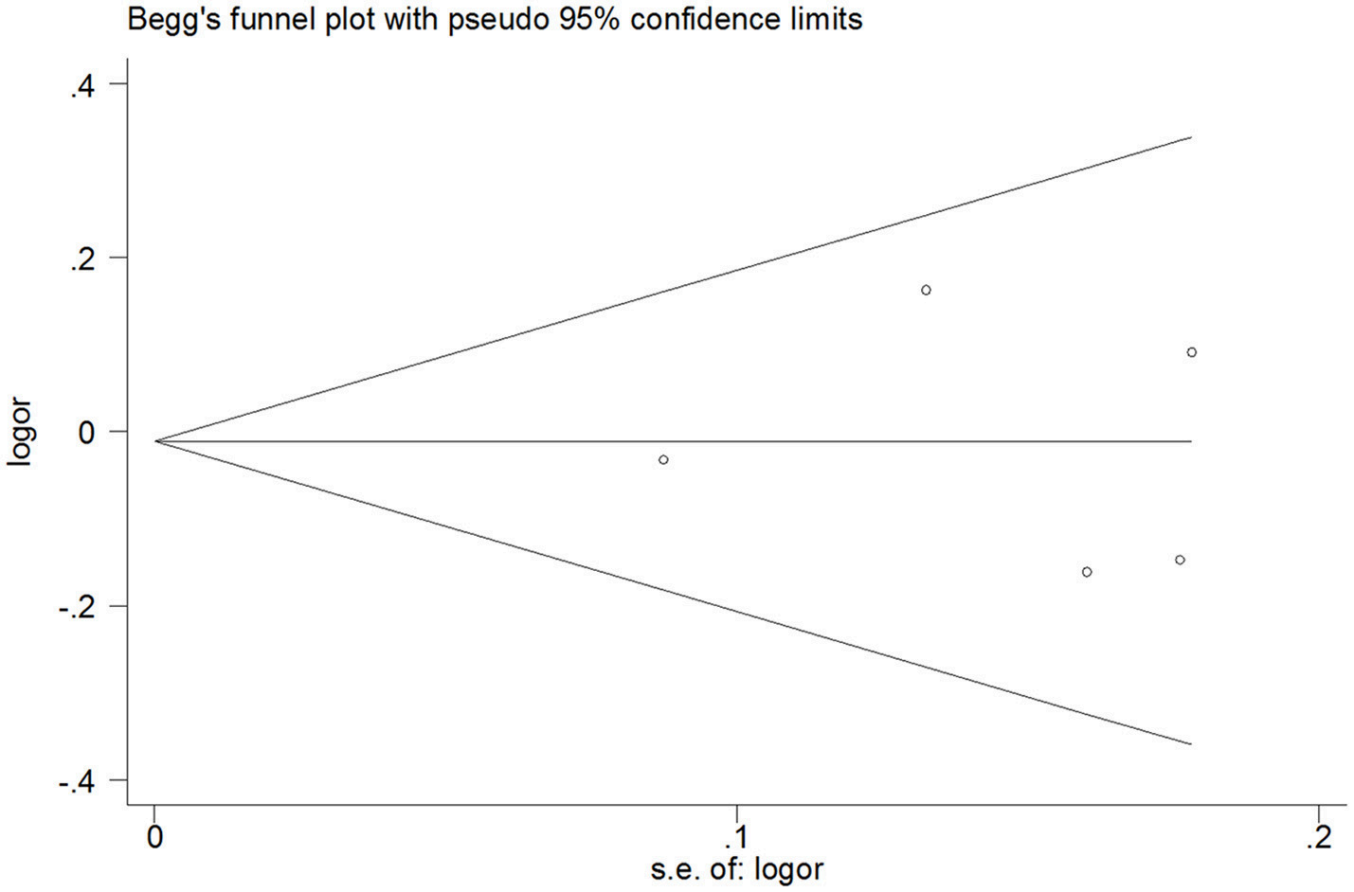
Begg's funnel plot of publication bias for heterozygous model of miR-146a C>G (rs2910164) polymorphism.

It is shown from Tables 2–5 that no significant publication bias was observed because the *P*-values for Egger's linear regression tests are no less than 0.05. Moreover, Begg's funnel plots did not show any significant asymmetry (Figure 6).

## Discussion

MiRNAs play key roles in many physiological and pathological processes, including tumorigenesis, proliferation, hematopoiesis, metabolism, immune function, epigenetics, and neurodegenerative diseases (Miska, 2005; O'Connell et al., 2010). SNP in miRNAs correlated closely with traditional risk factors of IS, such as atherosclerosis, hypertension, diabetes and hyperlipidemia (high cholesterol) (Boucherat et al., 2015; Li et al., 2015; Gómez-Banoy and Mockus, 2016; Schober and Weber, 2016). The four miRNAs we focused on—miR-146a, miR-149, miR-196a2, and miR-499—are intimately related to regulation of TNF-α (El Gazzar et al., 2011), MTHFR (Wu et al., 2013), annexin A1 (ANXA1) (Luthra et al., 2008), and CRP (Yang et al., 2012) respectively, which were general causes of cerebral ischemia. Above all, these miRNAs might be associated with risk of IS.

In the present meta-analysis, we found that the miR-499 A>G (rs3746444) G allele presented 1.24-fold higher risk of IS than A allele, as well as the AG and AG + GG carriers presented 1.35- and 1.36-fold higher risk than AA carriers, respectively. Previous researches have revealed that miR-499 involved in cell death under the anoxia and ischemia condition through its suppression of calcineurin-mediated dephosphorylation of dynamin-related protein-1 (Drp1), as a result the Drp1 accumulation in mitochondria and the Drp1-mediated activation of the mitochondrial fission machinery reduced (Wang et al., 2011). MiR-499 activates the production of CRP and regulates the expression of many inflammatory cytokines, including IL-17RB, IL-23a, IL-2R, IL-6, IL-2 and IL-18R, so that it is able to influence the inflammatory reaction (Yang et al., 2012; Hashemi et al., 2013). Its increase of the concentration payed on serum or plasma CPR is likely to cause the increase of the incidence of stroke through regulating the level of risk factors of IS, such as in ammation, hypertension, and hyperlipoidemia (Luo et al., 2017). Liu et al. (2014) found the association between increased IS risk and rs3746444 A/G variant genotypes (GG + AG) was more notable in younger subjects, never smokers, non-obesity (BMI ≤ 24), non-hypertension non-diabetes, non-coronary heart disease and non-hyperlipidemia. The results are inconsistent and its other potential mechanisms are still uncertain.

There was no significant association between miR-146a C>G (rs2910164) and IS risk in the overall analyses. According to the previous studies, miR-146a inhibits interleukin-1 receptor-associated kinase 1 (IRAK-1) and tumor necrosis factor receptor associated factor 6 (TRAF-6) expression through negative feedback regulation (Ramkaran et al., 2014), causing decreased levels of pro-inflammatory cytokines and critical transcription factor in atherosclerosis such as IL-1, IL-6, IL-8, TNF-α, and resulting inhibition of nuclear factor (NF)-κB via the Toll-like receptor pathway (Guo et al., 2010). Therefore, the down-regulation of miR-146a tends to reduce inflammation-related atherosclerosis and have an effect on vascular damage response. The polymorphisms of miRNA will affect the further processing of pre-miRNA into a mature miRNA, therefore influence occurrence and development of disease (Saunders et al., 2007). Furthermore, miR-146a G allele may decrease the level of mature miRNA expression (Shen et al., 2008). Zhu et al. (2014) found that miR-146a CC genotype and C allele are associated with an increased incidence of large-artery atherosclerotic stroke in the northern Chinese Han population. However, Qu et al. (2016) conducted a study with 1,311 patients in six provinces and found rs2910164 has no association with IS incidence under several genetic models, stroke subtype and sex. It appears to be a strong predictor of stroke prognosis but not stroke incidence. Further reasons for the different results may include: the difference of geographical factors, sample size, study design, selection criteria of the patients or the diversity of genotyping methods, as well as not all studies obtained their results after adjustment of conflicting factors. More explorations in more diverse geographically regions with large sample size are expected to confirm the conclusions.

The present meta-analysis did not find association between miR-149 T>C (rs2292832), miR-196a2 T>C (rs11614913), and IS risk under all genetic models. In fact, no study has elucidated that the genetic polymorphism of miR-196a2 T>C (rs11614913) is associated with stroke. For miR-149 T>C (rs2292832), it might be spurious result because the results were on basis of only one study with limited participants. Whether miR-149 T>C (rs2292832), miR-196a2 T>C (rs11614913), are related to IS risk or not is an interesting question for future investigation. Furthermore, the two SNPs mentioned have been demonstrated to be related to other human diseases, including cancer (Hu et al., 2008), congenital heart disease (Xu et al., 2009) and coronary heart disease (Sung et al., 2016), or correlated with IS when they are combined with other SNPs, suggesting that these polymorphisms tend to influence different human diseases in various ways as well as reflecting different genetic or etiological factors for different diseases.

In addition, some studies explored the gene–gene, gene-environment interaction between miRNA polymorphism and IS risk. Jeon et al. (2013) constructed all possible allele combinations and found miR-146a G/-149 T/-196a2 C/-499 G, miR-146a C/-196a2 T/-499 A, miR-146a C/-196a2 T were significantly associated with disease prevalence in Korea. Huang et al. (2015) focused on the combined effects between the stroke-associated polymorphism (miR-146a) and fasting glucose, HDL-c, and LDL-c levels on the risk of IS and found that the combined effects between miRNA polymorphism and fasting glucose/blood lipid levels may contribute to stroke pathogenesis.

To our knowledge, this is the first meta-analysis that evaluates the association between these four polymorphisms and risk of IS in Chinese. Sensitivity analysis by HWE status of controls made it more precise. Therefore, our findings were more reliable than the conclusions of previous studies. These results might provide further implications for evaluating risk of IS.

However, the limitations in our meta-analysis shouldn't be ignored when interpreting our findings. First of all, the evidence of between study heterogeneity was apparent, which might distort the conclusion of this meta-analysis. Second, the objects in all included studies were totally enrolled from hospitals, which might not be sufficient to represent the huge Chinese population and widespread distribution nationwide. Thirdly, IS is a complicated and multifactorial disease influenced by both genetic and environment factors. But the effects of the combination of genetic and environmental factors are difficult to be detected due to the lack of the accurate individual information in studies included. Lastly, a SNP might be in linkage disequilibrium with other genetic variations of stroke susceptibility genes, which may present stronger effect when considered together with other variations.

In conclusion, miR-499 A>G (rs3746444) G allele and AG, AG + AA genotype might be risk factors of IS in Chinese. No significant association was observed between miR-146a C>G (rs2910164), miR-149 T>C (rs2292832), miR-196a2 T>C (rs11614913) polymorphism and risk of IS. The associations may be different due to geographical factors of China. More explorations in more diverse geographically regions with large sample size are expected to further verify the findings in the future.

## Author contributions

W-JL and C-XL: planned and designed the study; HW, C-XL, and JZ: searched the databases and extracted the data; C-XL, HW, and Z-HF: performed statistical analysis; C-XL: drafted the manuscript; W-JL and J-LO: reviewed the manuscript. All authors approved the final paper for submission and publication.

### Conflict of interest statement

The authors declare that the research was conducted in the absence of any commercial or financial relationships that could be construed as a potential conflict of interest.

## References

[B1] Bartel D. P. (2009). MicroRNA target recognition and regulatory functions. Cell 136, 215–233. 10.1016/j.cell.2009.01.002 19167326 PMC3794896

[B2] Boehme A. K. Esenwa C. Elkind M. S.. (2017). Stroke risk factors, genetics, and prevention. Circ. Res. 120, 472–495. 10.1161/CIRCRESAHA.116.308398 28154098 PMC5321635

[B3] Boucherat O. Potus F. Bonnet S. (2015). MicroRNA and pulmonary hypertension, in microRNA: Medical Evidence: From Molecular Biology to Clinical Practice, ed Santulli G. (Cham: Springer International Publishing), 237–252.

[B4] Duan R. Pak C. Jin P. (2007). Single nucleotide polymorphism associated with mature miR-125a alters the processing of pri-miRNA. Hum. Mol. Genet. 16, 1124–1131. 10.1093/hmg/ddm062 17400653

[B5] El Gazzar M. Church A. Liu T. McCall C. (2011). MicroRNA-146a regulates both transcription silencing and translation disruption of TNF-α during TLR4-induced gene reprogramming. J. Leukoc. Biol. 90, 509–519. 10.1189/jlb.0211074 21562054 PMC3157899

[B6] GBD Neurological Disorders Collaborator Group (2017). Global, regional, and national burden of neurological disorders during 1990-2015: a systematic analysis for the Global Burden of Disease Study 2015. Lancet Neurol. 16, 877–897. 10.1016/S1474-4422(17)30299-5 28931491 PMC5641502

[B7] Gómez-Banoy N. Mockus I. (2016). [MicroRNAs: circulating biomarkers in type 2 Diabetes Mellitus and physical exercise]. Rev. Med. Chil. 144, 355–363. 10.4067/S0034-98872016000300011 27299822

[B8] Guo M. Mao X. Ji Q. Lang M. Li S. Peng Y.. (2010). MiR-146a in PBMCs modulates Th1 function in patients with acute coronary syndrome. Immunol. Cell Biol. 88, 555–564. 10.1038/icb.2010.16 20195282

[B9] Hashemi M. Eskandari-Nasab E. Zakeri Z. Atabaki M. Bahari G. Jahantigh M. (2013). Association of pre-miRNA-146a rs2910164 and pre-miRNA-499 rs3746444 polymorphisms and susceptibility to rheumatoid arthritis. Mol. Med. Rep. 7, 287–291. 10.3892/mmr.2012.1176 23138379

[B10] Higgins J. (2008). Commentary: heterogeneity in meta-analysis should be expected and appropriately quantified. Int. J. Epidemiol. 37, 1158–1160. 10.1093/ije/dyn204 18832388

[B11] Hu Z. Chen J. Tian T. Zhou X. Gu H. Xu L.. (2008). Genetic variants of miRNA sequences and non-small cell lung cancer survival. J. Clin. Invest. 118, 2600–2608. 10.1172/JCI34934 18521189 PMC2402113

[B12] Huang S. Zhou S. Zhang Y. Lv Z. Li S. Xie C.. (2015). Association of the genetic polymorphisms in pre-MicroRNAs with risk of ischemic stroke in a Chinese population. PLoS ONE 10:e0117007. 10.1371/journal.pone.0117007 25658319 PMC4319971

[B13] Jeon Y. J. Choi Y. S. Rah H. Kim S. Y. Choi D. H. Cha S. H.. (2012). Association study of microRNA polymorphisms with risk of idiopathic recurrent spontaneous abortion in Korean women. Gene 494, 168–173. 10.1016/j.gene.2011.12.026 22222140

[B14] Jeon Y. J. Kim O. J. Kim S. Oh S. H. Oh D. Kim O. J.. (2013). Association of the miR-146a, miR-149, miR-196a2, and miR-499 polymorphisms with ischemic stroke and silent brain infarction risk. Arterioscler. Thromb. Vasc. Biol. 33, 420–430. 10.1161/ATVBAHA.112.300251 23202363

[B15] Kotlega D. Peda B. Zembron-Łacny A. Gołab-Janowska M. Nowacki P. (2017). The role of brain-derived neurotrophic factor and its single nucleotide polymorphisms in stroke patients. Neurol. Neurochir. Pol. 51, 240–246. 10.1016/j.pjnns.2017.02.008 28291539

[B16] Li T. Yang G. M. Zhu Y. Wu Y. Chen X. Y. Lan D.. (2015). Diabetes and hyperlipidemia induce dysfunction of VSMCs: contribution of the metabolic inflammation/miRNA pathway. Am. J. Physiol. Endocrinol. Metab. 308, E257–E269. 10.1152/ajpendo.00348.2014 25425000

[B17] Liu Y. Ma Y. Zhang B. Wang S. Wang X. Yu J. (2014). Genetic Polymorphisms in pre-microRNAs and risk of ischemic stroke in a Chinese population. J. Mol. Neurosci. 52, 473–480. 10.1007/s12031-013-0152-z 24178064

[B18] Luo H. C. Luo Q. S. Wang C. F. Lei M. Li B. L. Wei Y. S. (2017). Association of miR-146a, miR-149, miR-196a2, miR-499 gene polymorphisms with ischemic stroke in a Chinese people. Oncotarget 8, 81295–81304. 10.18632/oncotarget.18333 29113388 PMC5655283

[B19] Luthra R. Singh R. R. Luthra M. G. Li Y. X. Hannah C. Romans A. M.. (2008). MicroRNA-196a targets annexin A1: a microRNA-mediated mechanism of annexin A1 downregulation in cancers. Oncogene 27, 6667–6678. 10.1038/onc.2008.256 18663355

[B20] Min K. T. Kim J. W. Jeon Y. J. Jang M. J. Chong S. Y. Oh D.. (2012). Association of the miR-146aC>G, 149C>T, 196a2C>T, and 499A>G polymorphisms with colorectal cancer in the Korean population. Mol. Carcinog. 51(Suppl. 1), E65–E73. 10.1002/mc.21849 22161766

[B21] Miska E. (2005). How microRNAs control cell division, differentiation and death. Curr. Opin. Genet. Dev. 15, 563–568. 10.1016/j.gde.2005.08.005 16099643

[B22] O'Connell R. Rao D. Chaudhuri A. Baltimore D. (2010). Physiological and pathological roles for microRNAs in the immune system. Nat. Rev. Immunol. 10, 111–122. 10.1038/nri2708 20098459

[B23] O'Donnell M. J. Xavier D. Liu L. Zhang H. Chin S. L. Rao-Melacini P.. (2010). Risk factors for ischaemic and intracerebral haemorrhagic stroke in 22 countries (the INTERSTROKE study): a case-control study. Lancet 376, 112–123. 10.1016/S0140-6736(10)60834-3 20561675

[B24] Pan Y. Chen W. Xu Y. Yi X. Han Y. Yang Q. (2017). Genetic polymorphisms and clopidogrel efficacy for acute ischemic stroke or transient ischemic attack: a systematic review and meta-analysis. Circulation 135, 21–33. 10.1161/CIRCULATIONAHA.116.024913 27806998

[B25] Park Y. S. Jeon Y. J. Lee B. E. Kim T. G. Choi J. U. Kim D. S.. (2012). Association of the miR-146aC>G, miR-196a2C>T, and miR-499A>G polymorphisms with moyamoya disease in the Korean population. Neurosci. Lett. 521, 71–75. 10.1016/j.neulet.2012.05.062 22659075

[B26] Qu J. Y. Xi J. Zhang Y. H. Zhang C. N. Song L. Song Y.. (2016). Association of the MicroRNA-146a SNP rs2910164 with ischemic stroke incidence and prognosis in a Chinese population. Int. J. Mol. Sci. 17:E660. 10.3390/ijms17050660 27164084 PMC4881486

[B27] Ramkaran P. Khan S. Phulukdaree A. Moodley D. Chuturgoon A. (2014). MiR-146a polymorphism influences levels of miR-146a, IRAK-1, and TRAF-6 in young patients with coronary artery disease. Cell Biochem. Biophys. 68, 259–266. 10.1007/s12013-013-9704-7 23794009

[B28] Saunders M. A. Liang H. Li W.-H. (2007). Human polymorphism at microRNAs and microRNA target sites. Proc. Natl. Acad. Sci. U.S.A. 104, 3300–3305. 10.1073/pnas.0611347104 17360642 PMC1805605

[B29] Schober A. Weber C. (2016). Mechanisms of MicroRNAs in atherosclerosis. Annu. Rev. Pathol. 11, 583–616. 10.1146/annurev-pathol-012615-044135 27193456

[B30] Shen J. Ambrosone C. B. DiCioccio R. A. Odunsi K. Lele S. B. Zhao H. (2008). A functional polymorphism in the miR-146a gene and age of familial breast/ovarian cancer diagnosis. Carcinogenesis 29, 1963–1966. 10.1093/carcin/bgn172 18660546

[B31] Sung J. Kim S. Yang W. Kim W. Moon J. Kim I. (2016). MiRNA polymorphisms (miR-146a, miR-149, miR-196a2 and miR-499) are associated with the risk of coronary artery disease. Mol. Med. Rep. 14, 2328–2342. 10.3892/mmr.2016.5495 27430349

[B32] Thakkinstian A. McElduff P. D'Este C. Duffy D. Attia J. (2005). A method for meta-analysis of molecular association studies. Stat. Med. 24, 1291–1306. 10.1002/sim.2010 15568190

[B33] Wang A. X. Xu B. Tong N. Chen S. Q. Yang Y. Zhang X. W.. (2012). Meta-analysis confirms that a common G/C variant in the pre-miR-146a gene contributes to cancer susceptibility and that ethnicity, gender and smoking status are risk factors. Genet. Mol. Res. 11, 3051–3062. 10.4238/2012.August.31.2 23007982

[B34] Wang J. X. Jiao J. Q. Li Q. Long B. Wang K. Liu J. P.. (2011). MiR-499 regulates mitochondrial dynamics by targeting calcineurin and dynamin-related protein-1. Nat. Med. 17, 71–78. 10.1038/nm.2282 21186368

[B35] Wu C. Gong Y. Sun A. Zhang Y. Zhang C. Zhang W.. (2013). The human MTHFR rs4846049 polymorphism increases coronary heart disease risk through modifying miRNA binding. Nutr. Metab. Cardiovasc. Dis. 23, 693–698. 10.1016/j.numecd.2012.02.009 22647417

[B36] Xiang Y. F. Guo J. Peng Y. Tan T. Huang H. Luo H. T.. (2017). Association of miR-21, miR-126 and miR-605 gene polymorphisms with ischemic stroke risk. Oncotarget 8, 95755–95763. 10.18632/oncotarget.21316 29221163 PMC5707057

[B37] Xu J. Hu Z. Xu Z. Gu H. Yi L. Cao H. (2009). Functional variant in microRNA-196a2 contributes to the susceptibility of congenital heart disease in a Chinese population. Hum. Mutat. 30, 1231–1236. 10.1002/humu.21044 19514064

[B38] Yang B. Chen J. Li Y. Zhang J. Li D. Huang Z.. (2012). Association of polymorphisms in pre-miRNA with inflammatory biomarkers in rheumatoid arthritis in the Chinese Han population. Hum. Immunol. 73, 101–106. 10.1016/j.humimm.2011.10.005 22019503

[B39] Zhu R. Liu X. He Z. Li Q. (2014). MiR-146a and miR-196a2 polymorphisms in patients with ischemic stroke in the northern Chinese Han population. Neurochem. Res. 39, 1709–1716. 10.1007/s11064-014-1364-5 24952884

